# The complete mitochondrial genome of *Niviventer sacer* (Rodentia: Muridae)

**DOI:** 10.1080/23802359.2022.2100289

**Published:** 2022-07-29

**Authors:** Xinghan Lin, Xiaoqing Sun, Guochen Zang, Yaoyao Li, Haotian Li, Yuchun Li

**Affiliations:** Marine College, Shandong University, Weihai, China

**Keywords:** *Niviventer sacer*, *Niviventer confucianus*, complete mitochondrial genome sequence

## Abstract

*Niviventer sacer* (Rodentia: Muridae) had been regarded as a subspecies of *N. confucianus*, i.e. *N. c. sacer*, and was raised as a distinct species recently by our laboratory. We sequenced the complete mitochondrial genome of *N. sacer* first and annotated the genome structure. The total length of the genome was 16,308 base pairs (bp) containing 13 protein-coding genes (PCGs), two ribosomal RNA genes (rRNAs), 22 transfer RNA genes (tRNAs), and a control region. We also constructed the phylogenetic tree by maximum-likelihood method and it demonstrated that *N. sacer* was the sister clade of *N. confucianus*.

*Niviventer sacer* (Thomas, 1908), belongs to the family Muridae, which is the endemic species of Shandong district, China, has been raised as a distinct species rather than as a subspecies of *N. confucianus* recently based on morphological characteristics, karyotyping, and molecular evidence (Li et al. 2020). However, until now, there are no reports about the complete mitochondrial sequence of *N. sacer*. In this study, we first sequenced and annotated the complete mitochondrial genome of *N. sacer* and constructed phylogenetic tree with 13 protein-coding genes (PCGs) of *N. confucianus* and other 15 species from the Murinae family. The information shown in this article may provide additional molecular evidence about the phylogenetic relationship between *N. sacer* and *N. confucianus*, and expedite the research of the molecular evolution of genus *Niviventer*.

The specimen of *N. sacer* was captured in Mountain Ai (37.411891°N, 120.774894°E), Shandong Province, China, which was deposited at the Marine College, Shandong University, Weihai, China (contact person Yuchun Li, email: li_yuchun@sdu.edu.cn) under the voucher number S4915. All animal sample collection protocols were approved by the Ethics Committee of Shandong University and complied with the current laws of China, and the specimen was handled in a manner consistent with the guidelines approved by the American Society of Mammalogists (Sikes 2016). Using the EasyPure Genomic DNA kit (TransGen Biotech Co., Ltd., Beijing, China), total genomic DNA was isolated from muscle tissue. Complete mitochondrial genome was amplified by polymerase chain reaction (PCR) with 18 pairs of primers (Supplementary information, Table S1) and the complete genome sequence composed of 18 PCR products sequenced via Sanger sequencing was annotated using the BioEdit 7.25 (Hall 1999) and MEGA 7.0.14 software (Kumar et al. 2016). The mitochondrial genomes of *N. confucianus* (GenBank accession MH283869) were used for primers design and gene annotation as template.

The complete mitochondrial genome of *N. sacer* that supports the findings of this study is openly available in GenBank of NCBI at https://www.ncbi.nlm.nih.gov/nuccore/MZ935252.1 under the accession number MZ935252 which was 16,308 bp in length and contains a set of 13 PCGs, two ribosomal RNA genes (rRNAs), 22 transfer RNA genes (tRNAs), and a control region. The composition of overall base is 33.9% for A, 28.5% for T, 25.0% for C, and 12.6% for G. The length of 13 PCGs was 11,402 bp, and all of which were in the heavy strand except for *ND6* that was in the light strand. The PCGs begin with ATG except for *ND1*, *ND2*, and *ND5* which started with GTG, ATA, and ATT. TAA stop codon was present in eight PCGs (*COX1*, *COX2*, *ATP8*, *ATP6*, *ND3*, *ND4L*, *ND5*, and *ND6*), but five PCGs (*ND1*, *ND2*, *COX3*, *ND4*, and *Cyt b*) terminated with incomplete stop codon T–.

To investigate phylogenetic relationships between *N. sacer* and other Murinae species, the nucleotide sequence data of 13 PCGs of *N. sacer* and other 14 Murinae species belonging to genus *Niviventer*, *Leopoldamys*, *Berylmys*, *Bandicota*, and *Rattus* were used for phylogenetic analyses by the maximum-likelihood method in RAxML v7 (Stamatakis 2006) with GTR + G+I model using MEGA 7.0.14 software (Kumar et al. 2016), *Apodemus agrarius* and *Mus musculus* were used as outgroups.

The phylogenetic tree constructed is shown in Figure 1. The result showed that *N. sacer* had the closest relationship with *N. confucianus*, and the two species were the sister clade to each other. Furthermore, our molecular information may enrich the existing molecular data and facilitate the molecular evolution investigation of *Niviventer* species.

**Figure 1.**
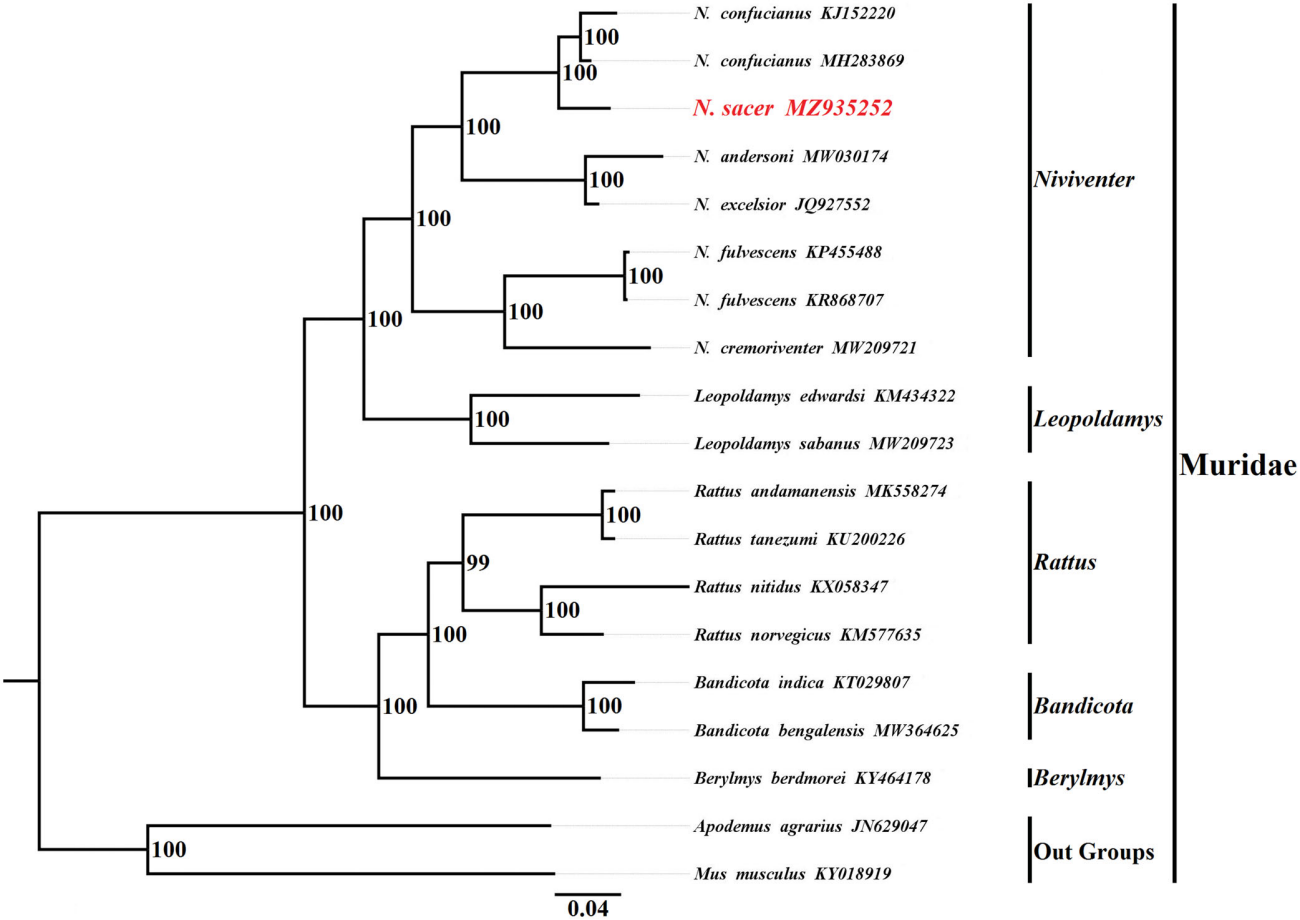
ML phylogenetic tree of 17 Murinae species based on the mitochondrial nucleotide sequence data of 13 protein-coding genes using GTR + G+I model. Nodal support was estimated by 1000 bootstrap replicates and ML bootstrap values are shown.

## Supplementary Material

Supplemental Material

## Data Availability

The genome sequence data that support the findings of this study are openly available in GenBank of NCBI at https://www.ncbi.nlm.nih.gov/nuccore/MZ935252.1 under the accession no. MZ935252.
