# Supplementary material for: The complete mitochondrial genome of Niviventer sacer (Rodentia: Muridae)
Source: Mitochondrial DNA B Resour. 2022 Jul 29;7(7):1369–71. doi: 10.1080/23802359.2022.2100289 (PMC9341373; doi:10.1080/23802359.2022.2100289)
Supplement: Supplemental Material [file TMDN_A_2100289_SM3031.doc]

**Table S1** PCR and sequencing primers used in the present study

| Amplified Fragment  Location | Primer Name | Nucleotide sequence 5′ to 3′ | Annealing Temperature | Citation |
| --- | --- | --- | --- | --- |
| 702~  1872 | NS1-F | TTACCTCACCACCTCTTGC | 54℃ | Current Study |
|  | NS1-R | GTGCTTGCCGAGTTCC |  |  |
| 1782~  2080 | NS1-2-F | GACAACTCGGATAACCATT | 52℃ | Current Study |
|  | NS1-2-R | TCGTTTAGCCATTCATTCT |  |  |
| 1948~  3128 | NS2-F | AGGAACTCGGCAAGCACG | 55℃ | Current Study |
|  | NS2-R | GATGGTTTGGGCGACAGC |  |  |
| 3051~  4078 | NS3-F | ATTCCCCTTCCATTACCC | 54℃ | Current Study |
|  | NS3-R | TTATTGAGGCTGTTGCTTGT |  |  |
| 3688~  4473 | NS4-F | ATTCTTCTTAGCGGGTATT | 47℃ | Current Study |
|  | NS4-R | TCATTCATCCTATGTGGG |  |  |
| 4306~  4691 | NS4-5-F | CCTAATCTTACTTATCTGACAAAAA | 54℃ | Current Study |
|  | NS4-5-R | GAAATCCTGTGAGGGGTG |  |  |
| 4501~  5519 | NS5-F | ATACTCCTCAATCGCCCACA | 58℃ | Current Study |
|  | NS5-R | AAGTCAGTTTCCGAAGCCAC |  |  |
| 5444~  6599 | NS6-F | TGAGCAGGAATAGTAGGAAC | 57℃ | Current Study |
|  | NS6-R | AGTGTAGGCATCTGGGTAG |  |  |
| 6419~  7499 | NS7-F | GCGGAGTAACTGGTATTG | 53℃ | Current Study |
|  | NS7-R | TTTAGACGACCTGGGATT |  |  |
| 7114~  8404 | NS8-F | TCCACGACCATACGCTAA | 57℃ | Current Study |
|  | NS8-R | CTCCGATTAGGTGTATTAGTAGAT |  |  |
| 8376~  9491 | NS9-F | AACACCCATCTCGCTTAT | 53℃ | Current Study |
|  | NS9-R | GGTTTATTTCAGGCAGTCA |  |  |
| 9160~  10552 | NS10-F | GGTTTTTACTTCACCATCCTCC | 51℃ | Current Study |
|  | NS10-R | CCAGCGTTTAGCCGTTCT |  |  |
| 10167~  11438 | NS11-F | ACGGAACCGACTATGTGC | 59℃ | Current Study |
|  | NS11-R | GCGGGATAATGTGACGAG |  |  |
| 11309~  12544 | NS12-F | AACTTTGCCCTTCCACCA | 54℃ | Current Study |
|  | NS12-R | AGATGCCTGCGACGACTA |  |  |
| 12195~  13558 | NS13-F | TCGGTTGGGAAGGAGTAG | 61℃ | Current Study |
|  | NS13-R | GTAGTGGCTGGGTGGTCT |  |  |
| 13323~  14605 | NS14-F | AAACCACCTCCAAACCCG | 60℃ | Current Study |
|  | NS14-R | CTTTATCTACTGAGAAACCGCCT |  |  |
| 14391~  15760 | NS15-F | ATTTCTGCCGAGCCGTAA | 55℃ | Current Study |
|  | NS15-R | ATTGATGCCCCTGAAGTAAG |  |  |
| 15717~  16308  1~795 | NS16-F | TCTACCATCCTCCGTGAA | 55℃ | Current Study |
|  | NS16-R | TACTAAATCCTCCTTTGTCCT |  |  |
